# Recent advances in targeting calcitonin gene-related peptide for the treatment of menstrual migraine: A narrative review

**DOI:** 10.1097/MD.0000000000029361

**Published:** 2022-06-17

**Authors:** Yan Jiang, Zhen-Lun Huang

**Affiliations:** Department of Encephalopathy, Traditional Chinese Hospital, Banan District, Chongqing, China.

**Keywords:** calcitonin gene-related peptide, CGRP receptors, menstrual migraine, treatment

## Abstract

Menstrual migraine (MM) has a longer duration and higher drug resistance than non-perimenstrual migraine. Calcitonin gene-related peptide (CGRP) and CGRP receptors are expressed in the peripheral and central nervous systems throughout the trigeminovascular system. The CGRP/CGRP receptor axis plays an important role in sensory physiology and pharmacology. CGRP receptor antagonists and anti-CGRP monoclonal antibodies (mAbs) have shown consistent efficacy and tolerability in the prevention of chronic or episodic migraine and are now approved for clinical use. However, few studies have reported the use of these drugs in MM, and no specific treatment for MM has been approved. This review aimed to shed light on the recent advances in targeting calcitonin gene-related peptides for the treatment of menstrual migraines in PubMed. In this review, we first discuss the axis of the CGRP/CGRP receptor. We then discuss the role of CGRP receptor antagonists and anti-CGRP mAbs in MM treatment. Finally, we discuss the role of the combination of anti-CGRP mAbs and CGRP receptor antagonists in migraine treatment and the drugs that inhibit CGRP release. Altogether, the anti-CGRP mAbs or CGRP receptor antagonists showed good efficacy and safety in the treatment of MM.

## 1 Introduction

Menstrual migraine (MM) has a longer duration and higher drug resistance than non-perimenstrual migraine. Patients with MM who do not respond to acute treatment may be suitable for either short-term or long-term preventive treatments.^[1]^ The drugs for short-term prevention of MM include triptans, cyclooxygenase-2 inhibitors, estrogen supplementation, and nonsteroidal anti-inflammatory drugs, which may delay rather than prevent attacks.^[1]^ Topiramate can be used for the long-term prevention of MM, which can reduce the frequency but not the duration or severity of perimenstrual attacks.^[2]^ It is contraindicated for hormonal contraceptives containing estrogens because of its limited effectiveness and increased risk of stroke.^[3–5]^ It is also contraindicated for women with migraine with or without aura, who are smokers and/or aged 35 or older treated with exogenous estrogens.^[6,7]^ So nonhormonal and long-term preventive treatments should be explored for MM.

Calcitonin gene-related peptide (CGRP) and CGRP receptors are expressed in the peripheral and central nervous systems throughout the trigeminovascular system. The CGRP/CGRP receptor axis plays an important role in sensory physiology and pharmacology. CGRP receptor antagonists and anti-CGRP monoclonal antibodies (mAbs) have shown consistent efficacy and tolerability in the prevention of chronic or episodic migraine and are now approved for clinical use.^[8–10]^ However, only a few studies have reported the use of these drugs in MM. Currently, no specific treatment for MM has been approved to date. Therefore, recent advances in targeting calcitonin gene-related peptides for the treatment of menstrual migraines were reviewed. In this review, we first discuss the axis of the CGRP/CGRP receptor. We then discuss the role of CGRP receptor antagonists and anti-CGRP mAbs in MM treatment. Finally, we discuss the role of the combination of anti-CGRP mAbs and CGRP receptor antagonists in migraine treatment and the drugs that inhibit CGRP release. This study did not involve the patients’ private information, so this review did not require ethical approval.

## 2 Methods

We searched for relevant articles in PubMed (date: January 1, 2016 to August 31, 2021) using the following terms: “menstrual migraine and calcitonin gene-related peptide” (N = 14), “menstrual migraine and CGRP receptor antagonist” (N = 3), “menstrual headache and calcitonin gene-related peptide” (N = 10), “menstrual headache and CGRP receptor antagonist” (N = 3), “perimenstrual migraine and calcitonin gene-related peptide” (n = 2), and “perimenstrual migraine and CGRP receptor antagonist” (n = 2). A total of 34 articles were retrieved from the database. We carefully reviewed the remaining articles and excluded overlapped same article, not accessible and no clinical case. Then, a total of 3 case reports (3492 patients) were used to analyze. It is not necessary for the approval of institutional review board because this is an evidence-based narrative review.

### 2.1 The axis of CGRP/CGRP receptor

CGRP is a peptide consist of 37-amino acid. It is one of the members of a peptide family includes CGRP and adrenomedullin (AM), calcitonin (CT), and amylin (AMY). αCGRP and βCGRP are the two forms of CGRP in humans.^[11,12]^ αCGRP is produced by alternate splicing of the *CT* gene, and βCGRP has a separate genetic origin. αCGRP is highly expressed in sensory neurons and βCGRP is expressed in the enteric nervous system.^[13]^

The CGRP receptor is a member of the family B G-protein-coupled receptors (GPCRs). The CGRP receptor includes a single transmembrane protein 1 (RAMP1) and a multimeric complex composed of seven transmembrane GPCRs with CT receptor-like receptor (CLR) domains. RAMPs include RAMP1, RAMP2, and RAMP3. CLR can partner with any one of the RAMPs and produces ligand specificity that interacts with a specific RAMP. CLR with RAMP1 forms a CGRP receptor CLR with RAMP2 or RAMP3 produces adrenomedullin AM1 and AM2 receptors, respectively. RAMPs can also form heteromers with CT receptors (CTRs). The forms of AMY receptors AMY1, AMY2, and AMY3 are CTRs linked to RAMP1, RAMP2, and RAMP3, respectively.^[14,15]^ A review has summarized the composition, agonist, and antagonist pharmacology of the calcitonin family of receptors.^[10]^

The 2-domain models were thought to be the main mechanism of CGPR binding to its receptor. In the first model, the C-terminal region of CGRP first binds to the extracellular N-terminal regions of CLR and RAMP1, forming an affinity trap. The N-terminus of CGRP interacts with the juxta-membrane region of CLR and triggers the accumulation of cAMP. In the second model, the binding of CGRP to the AMY1 receptor (CTR + RAMP1) though amino acids shared between CLR and CTR, which interact with RAMP1 residue tryptophan 84.^[16]^

CGRP and CGRP receptors are expressed in the peripheral and central nervous systems throughout the trigeminovascular system. The CGRP/CGRP receptor axis plays an important role in sensory physiology and pharmacology.^[17]^ CGRP is a potent vasodilator and mediator of pain signal transmission after activation of trigeminal sensory nerve fibers. The concentration of CGRP increased in the external jugular venous blood during a migraine attack compared to that during a non-migraine attack. The CGRP level was reduced with migraine relief by treatment. These findings support the potential role of CGRP in migraine.^[18]^ However, CGRP may prolong activation of trigeminal pathways and cause episodic migraine into chronic migraine, which leads to fewer treatment options.^[19]^ Therefore, blocking the axis of the CGRP/CGRP receptor could be a possible treatment for migraine.

### 2.2 CGRP receptor antagonists in MM treatment

CGRP receptor antagonists (CGRP-RAs) can block the initial CGRP peptide binding event and subsequent receptor activation by binding to CGRP receptors through a hydrophobic pocket formed by CLR and RAMP 1.^[20]^ In the clinic, olcegepant (BIBN4096BS) as the first CGRP-RA can completely block both CGRP and AMY1 receptors.^[21]^ All the next-generation oral CGRP-RAs including telcagepant (MK-0974), MK-3207), rimegepant (BMS-927711), ubrogepant (MK-1602), and atogepant (AGN-241689): MK-8031 have activity against the AMY1 receptor at therapeutic plasma concentrations. The olcegepant (BIBN4096BS) also has antagonist activity of the CGRP receptor, which is less selective for the CGRP receptor than commonly reported.^[22]^

The olcegepant (BIBN4096BS) is the first non-peptide CGRP-RA that develops and tests in humans and is effective in the acute treatment of migraine.^[23]^ However, olcegepant can only be used as an acute antimigraine therapeutic and is poorly absorbed after oral administration because of its high molecular weight and high polarity with several H-bond donors, which leads to limited further development. Small-molecule oral CGRP-RAs have been developed and shown to be effective against migraine.^[24–28]^ These CGRP-RAs included telcagepant (MK-0974, MK-3207, rimegepant (BMS927711), BI-44370TA, ubrogepant, and atogepant.

It has been extensively studied on migraine pain mechanisms, potential benefits, and limitations of CGRP modulation in the acute treatment of migraine for telcagepants.^[29]^ The Telcagepant was also used as a headache prophylaxis with MM. Patients with MM were randomized to receive telcagepant or placebo in a 2:1 ratio to evaluate the safety and efficacy of headache prophylaxis.^[30]^ A telcagent with a dose of 140 mg was used for seven consecutive days peri-menstrually. This study showed that telcagepants cannot reduce the monthly headache frequency. However, it can reduce the number of days of perimenstrual headaches. Approximately 2.5% and 2.7% of patients were discontinued because of adverse events for the telcagent and placebo, respectively.

However, many small molecule CGRP-RAs in registration were halted by safety with unacceptable drug-induced liver injury (DILI).^[25]^ The hepatotoxic effects of telcagepant were not observed with intermittent use for 18 months for the acute treatment of migraine.^[31]^ However, hepatotoxic effects can develop in chronic or when intensive use for migraine prevention or MM.^[32–35]^

At present, only a few reports have shown CGRP-RAs in the treatment of MM. Therefore, further studies should explore the role of CGRP-RAs in the treatment of MM. Toxicity should also be studied.

### 2.3 Anti-CGRP mAbs in MM treatment

Small oral molecules are generally preferred for acute treatment. However, antibodies have some important advantages over small-molecule drugs, especially for chronic treatment because of their long-circulating plasma half-lives (weeks) and low toxicity. The anti-CGRP mAbs as potential migraine therapeutics by early human experimental research could nevertheless induce a migraine attack.^[18]^ However, some studies have shown that anti-CGRP mAbs are effective for treating migraine and CGRP-induced headaches.^[23,36]^ There are four CGRP mAbs that have been approved by FDA and showed efficacy in the prevention of frequent episodic and chronic migraine. These approved CGRP mAbs included galcanezumab, erenumab, eptinezumab, and fremanezumab in episodic migraine and chronic migraine.^[8,9]^

Data on the role of anti-CGRP mAbs in MM are rare. Recently, a report compared the difference between menstrual and non-menstrual women with chronic migraine with erenumab treatment.^[37]^ Total of 18 women with 11 erenumab responders and 7 erenumab non-responders were enrolled in this study. A total of 103 menstrual cycles and 2926 days were observed. The results showed that, for responders or non-responders, the proportion of headache days was higher on menstrual days than on premenstrual/non-menstrual days. Similarly, it was higher on menstrual days than on premenstrual or non-menstrual days with erenumab non-responders. Therefore, even when treated with erenumab, migraine is more frequent than outside menstrual days.

Another study on the efficacy and safety of erenumab in the prevention of MM has also been reported.^[38]^ The patients were divided into three groups: placebo, erenumab 70 mg, and erenumab 140 mg. The drugs were administered subcutaneously once monthly for the 6-month. Monthly migraine days include perimenstrual and intermenstrual migraine attacks. The monthly acute migraine-specific medication days were significantly decreased in the erenumab 70 and 140 mg groups than in the placebo group. Similar adverse events were observed in all the groups without cardiovascular events. Therefore, it is safe and effective for the prevention of MM with erenumab. However, more studies should be performed to explore the efficacy and safety of anti-CGRP mAbs in the prevention of MM.

### 2.4 Combination of anti-CGRP mAbs and CGRP receptor antagonists

Both anti-CGRP mAbs and CGRP receptor antagonists play an important role in the preventive and acute treatment of headache. Treatment of breakthrough attacks during preventive treatments is necessary. Therefore, the use of a combination of anti-CGRP mAbs and CGRP receptor antagonists may result in better outcomes. Recently, two patients with migraine were treated with CGRP receptor antagonists (rimegepant) and anti-CGRP mAbs (erenumab). The results showed that rimegepants are effective for migraine attacks that occur during preventive erenumab therapy. Erenumab is also effective for migraine prevention during the coadministration of rimegepants for acute treatment.^[39]^ Another report showed 13 patients with migraine who simultaneously used rimegepant, erenumab, fremanezumab, or galcanezumab to evaluate the safety and tolerability of the co-administered drugs.^[40]^ The 13 patients were treated combined with 7 erenumab, 4 fremanezumab, and 2 galcanezumab. The results showed that no serious adverse effects were observed. Therefore, the combination of anti-CGRP mAbs and CGRP receptor antagonists is effective and safe. However, the mechanism underlying the benefits of concomitant use of anti-CGRP mAbs and CGRP receptor antagonists is unknown and requires further study.

### 2.5 Drugs inhibits CGRP release

5-HT agonists can suppress CGRP release in preclinical studies. The ergots and 5-HT1B/5-HT1D receptor agonists (triptans) can attenuate elevated levels of CGRP.^[41–43]^ Pharmacological experiments have shown that triptans inhibit CGRP release through action at prejunctional 5-HT1D receptors.^[44–47]^ These studies led to the initiation of novel antimigraine drug discovery programs targeting CGRP and its receptor.^[48]^ Therefore, the combination of anti-CGRP mAbs or CGRP receptor antagonists with drugs that inhibit CGRP release may have better outcomes in the treatment of MM.

## 3 Conclusion

Altogether, the anti-CGRP mAbs or CGRP receptor antagonists showed good efficacy and safety in the treatment of MM. However, a larger number of prospective and multicenter studies are needed to further explore the role of anti-CGRP mAbs or CGRP receptor antagonists in the treatment of MM. In addition, the role of the combination of anti-CGRP mAbs with CGRP receptor antagonists in the treatment of MM is also needed for further research in clinics and on the mechanism underlying the benefits of concomitant use. Finally, the efficacy and safety of the combination of anti-CGRP mAbs or CGRP receptor antagonists with drugs that inhibit CGRP release should be explored in the treatment of MM.

## Acknowledgments

The funders had no role in study design, data collection and analysis, decision to publish, or preparation of the manuscript.

## Author contributions

All the authors wrote and approved the paper.

**Conceptualization:** Yan Jiang, Zhen-Lun Huang.

**Data curation:** Yan Jiang, Zhen-Lun Huang.

**Investigation:** Yan Jiang, Zhen-Lun Huang.

**Writing – original draft:** Yan Jiang.

**Writing – review & editing:** Yan Jiang, Zhen-Lun Huang.
